# Effects of Mining Activities on the Release of Heavy Metals (HMs) in a Typical Mountain Headwater Region, the Qinghai-Tibet Plateau in China

**DOI:** 10.3390/ijerph15091987

**Published:** 2018-09-12

**Authors:** Wenhao Wei, Rui Ma, Ziyong Sun, Aiguo Zhou, Jianwei Bu, Xiang Long, Yunde Liu

**Affiliations:** 1 Geological Survey, China University of Geosciences, Wuhan 430074, China; weiwenhao048@cug.edu.cn; 2 School of Environmental Studies, China University of Geosciences, Wuhan 430074, China; ziyong.sun@cug.edu.cn (Z.S.); aiguozhou@cug.edu.cn (A.Z.); jangous@163.com (J.B.); longxiang@cug.edu.cn (X.L.); lydcn84@126.com (Y.L.); 3 Laboratory of Basin Hydrology and Wetland Eco-restoration, China University of Geosciences, Wuhan 430074, China

**Keywords:** heavy metals, mining activity, water quality, headwater region

## Abstract

Understanding the heavy metal (HM) contamination in alpine mountain headwaters regions is important to maintaining the ecosystem stability of the basin. A total of 119 water samples and 104 sediment samples were collected along tributaries and the main course of Heihe River. The concentrations of eight heavy metals (As, Cd, Cr, Cu, Mn, Ni, Pb, and Zn) in water and sediment were measured to describe their spatial variability and to assess water quality. To identify the origins and pathways of HMs, anions, cations, and trace elements, as well as *δ*D/*δ*^18^O stable isotopes in water samples were also measured. The results of water quality assessment suggested that tributaries were affected by local mining activity. Factor analysis in sediments showed that all HMs in sediments were inherited from the parent bedrock. Both natural weathering and mining contribute HMs. Cr and Ni were homologous with a source from the weathering of basic gabbro and serpentine at Yushigou. Mn appeared to be influenced more by artificial activities such as agriculture and grazing. Depending on the mining technique involved, two pathways for the release of HMs were distinguished in this area. For open-pit mining, mining promoted the release of HMs primarily via enhanced weathering. For underground mining, HMs might have contributed to greater acid mine discharge at high elevations due to the weak weathering processes. As the elevation decreases, precipitation increases, and a series of complex hydrological factor significantly affect leaching and runoff. The study results can be applied to improve water management efficiency.

## 1. Introduction

Heavy metal (HM) contamination in water environments has received considerable attention worldwide due to the toxicity, persistence, abundance, and biomagnification of HMs in the environment and their subsequent accumulation in aquatic habitats [1,2]. HMs in water environments originate from multiple sources, including natural processes such as volcanism, bedrock weathering, erosion and anthropogenic activities, such as industry, agriculture fertilization and drainage, and especially mining, metal smelting and refining [1,2,3,4]. Many previous studies have shown that the most critical contributor to HM pollution in river basins is mining activity [1,5]. Understanding the concentration, distribution and the sources of HMs in aquatic environments is essential to provide a scientific reference for the protection of water resources and the control of water pollution.

Like other alpine mountains around the world (e.g., the Alps and Rocky Mountains and the Tibetan Plateau), the headwaters region of the Heihe River basin, often referred as a “water tower”, plays an essential role in maintaining the ecosystem stability of the basin. The surface runoff in upstream headwaters contributes approximately 90% of the water resources in the midstream and downstream areas [6]. In addition, anthropogenic activities such as the expansion of artificial oases, large-scale agricultural development and overuse of synthetic substances in midstream and downstream areas have increased the dependence of the quantity and quality of water resources on upstream headwater regions [6,7].

The headwaters region of the Heihe River basin has gained considerable attention due to the special geological and metallogenic conditions. With the Western Development Initiative (WDI) launched in 2002, the Chinese government has established 22 mineral exploration plan areas on the Qinghai-Tibet Plateau [8], where the Qilian-Tianjun coal, copper, lead, and zinc planning area is located. A large number of mines have been placed into operation since the 1980s. Unsustainable mineral exploration and mining activity have severely damaged the fragile local ecological environment and compromised the water quality [9]. Additionally, some mines are located near glaciers at high elevations [10]. Therefore, the quality of the meltwater, which is the vital source of water for the river, may be deteriorated by mining activity. This area has increasingly drawn the attention of local and central governments [11]. However, many researchers are concerned with quantifying surface runoff to serve for basin water resource allocation [12,13,14,15,16,17,18]. Little attention has been paid to the deterioration of the water quality in response to human activities such as mining.

The present study aimed to investigate the contents of As, Cd, Cr, Cu, Ni, Pb, and Zn in water environments across the headwater region of the Heihe River and to determine their regional variability and their natural or artificial origin. By analyzing the spatial distribution patterns of HM elements in surface water, we identify the sources of the HMs, and evaluate the surface water quality. The results can be applied to improve water management efficiency.

## 2. Materials and Methods

### 2.1. Study Area

**Figure 1:**
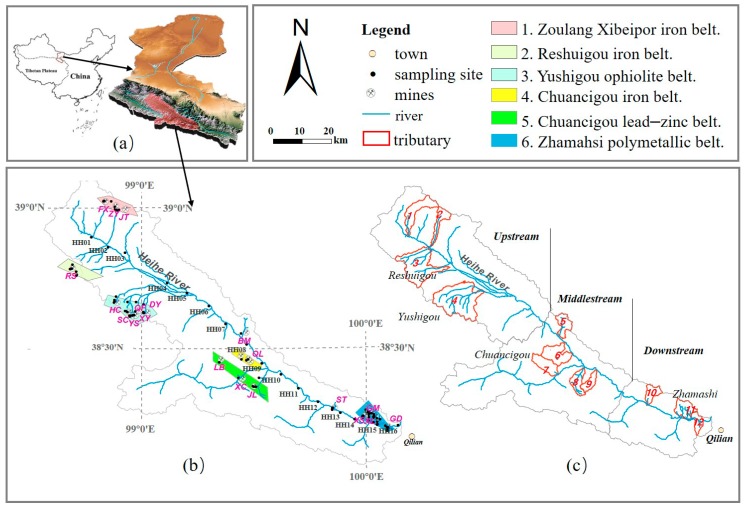
(**a**) Location of the headwaters region of the Heihe River basin. (**b**) A map of the sampling sites, including 6 ore concentration areas and 22 mines. The Fengxin (FX); Xiaoshalongzhigou (JT), Xiaoshuigou (ZY); Reshuigou (RS), Shuangchagou (SC), Heicigou (HC), Yushigou (YS), Dongyushigou (DY), and Qifeng (QF) located at the upstream of this study area. The Bianmagou(BM), Xiaoshuigou (QL), Liaobantai (LB), Xinchuan (XC), Daerzhulong (JL) in middle-stream. And Shitougou (ST), Wanyanghe (WY), Xialiugou (XL), Xishanliang (XS), Guomisi (GM), and Xiagou (XG) and Donggou (DG) in downstream area. (**c**) A map of the investigated tributaries and upstream, middle-stream, and downstream; The number in red within different closed shapes indexes the different tributary. Further detailed information on the tributaries and the mines is shown in Table 1.

The headwaters region of the Heihe River basin is located at the northern margin of the Qinghai-Tibet Plateau in the Qilian Mountains of Qinghai Province at 39°51′–38°91′ N and 98°34′–100°11′ E (Figure 1) with a basin area of 5037.38 km^2^. The main landform is alpine valleys, and the average elevation ranges from 2583 to 4891 m above mean sea level (m.s.l). The region has a continental alpine climate with a mean annual air temperature of 1 °C and mean annual precipitation of 420 mm, which mainly occurs in summer. The length of the Heihe River in the study area is more than 175 km, extending from Tieli Mountain through Bayi Glacier to Huangzang Temple. There is apparent vertical zoning in the vegetation landscape.

#### 2.1.1. Geology

The headwaters region of the Heihe River belongs to the northern Qilian orogenic belt, which is rich in ores with various nonferrous metals, ferrous metals and nonmetallic building materials [9]. This belt is an NW–SE-trending oceanic suture belt that lies between the Alashan Block and the Qilian Block. The belt is composed of subduction accretionary complexes, including Neoproterozoic to early Paleozoic ophiolite sequences, high-pressure metamorphic rocks, island arc volcanic rocks and granites, and post-Devonian sedimentary cover sequences [19,20].

#### 2.1.2. Anthropogenic Activity

In this typical alpine mountain region, the main anthropogenic activities consist of agricultural, grazing and mining activities. The traditional agriculture in this area has been greatly limited by the cold climate. Town and cropland near the main course of Heihe River were used as sites for the study of agricultural activity, while the grazing activity in this area were usual for wide alpine meadow pastures [21] (shown in Figure S2).

In the study area, the early Paleozoic was the main metallogenic period. The principal deposit combinations are Cu–Zn, Cu–Pb–Zn, Cu–Ni, and Cr. The large-scale mineralizations that formed mineable metals during this period were sulfide deposits. Previous studies showed that the formation of metal sulfide deposits was related to submarine volcanism and subduction orogenic processes [22,23,24]. There are six ore concentration areas in the different reaches of this basin. There are two iron ore belts in this area, and both of them are metamorphic hydrothermal sedimentary deposits. The Zoulang Xibeipor iron belt is located on the north bank of Heihe River and contains the Xiaoshalongzhigou (JT) and Xiaoshuigou (ZY) deposits. Moreover, the northern Tolle Mountain iron ore belt is located on the south bank of the Heihe River and contains RS and JT iron ore. These ores are produced underground. Both the Yushigou and Chuancigou areas with complete ophiolite sequences [19,22,23,25] are on the south side of the Heihe River. The Yushigou area is a copper–lead–zinc–chromium metallogenic deposit belt, including Shuangchagou (SC), Heicigou (HC), Yushigou (YS), Dongyushigou (DY), and Qifeng (QF) deposits. During the investigation, only open-pit mining was found in this area. In the Chuancigou area and Zhamashi area, the copper polymetallic deposits developed in acid island arc volcanic rocks and high-pressure rocks such as blueschist [19], and the Wanyanghe (WY), Xialiugou (XL), Xishanliang (XS), Guomisi (GM), and Xiagou (XG) deposits are located at these areas. These deposits are often coincident with iron and manganese deposits in particular. Underground mining technology has been widely applied in the study area.

### 2.2. Water Sample Collection and Analysis

To assess the water quality of this study area and to identify the source and pathway of the HMs releasing, a total of 119 sets of water samples were collected from and the 22 mines and the main course of Heihe River in August 2013. Figure 1 and Table 1 show all sampling sites and the distribution of mines and tributary catchments, with serval mines for along each tributary. Several mines are located along the upstream tributaries catchment near the glacier. Fifty-eight river water samples were collected in the surface stream around the mines; 10 meltwater samples were collected in several tributaries’ headwater streams near the glacier. Seven groundwater samples were collected, including two spring water samples and five samples from drinking water wells. Moreover, six pond water samples were collected around ore slag heap, and 22 samples of mineral leached water were collected from underground mines and opencast dumps, one of which was collected from a tailings pond in the Xinchuan (XC) concentrator. In the main course of the Heihe River, 16 river water samples (HH01-HH16) were collected near the discharge point of those tributaries to determine their contributions to the HMs in the corresponding tributaries (shown in Figure 1).

All water samples were filtered with 0.22 μm membranes in the field into polythene bottles that had been thoroughly prewashed with deionized water. During the sampling times, parameters such as pH and electrical conductivity were measured on site using a portable Hatch Ec and pH meter (HACH HQ40d, Loveland, CO, USA) with the calibration of pH 4, pH 7 and pH 9 standards, and alkalinity (HCO_3_^−^ and CO_3_^2−^) was determined on the sampling day using the Gran titration method, which determines the equivalent potentiometric point via titration [26] within 12 h of sampling. Samples for cation and trace element analysis were acidified with ultrapure HNO_3_ to pH = 2 and stored at 4 °C until being shipped to the laboratory [27] for ion analysis, including the analysis of HMs. To understand the hydrological process using isotope analysis, 99 water samples were collected from different source, including surface stream, pond, underground (well/spring), and mineral leached water. Twenty samples (including 19 mineral leached water samples and one groundwater sample) were abandoned since these samples had high total dissolved solid (TDS) values (greater than 500 mg/L).

Anion, cation, minor element, and isotope analyses were all performed in the Laboratory of Basin Hydrology and Wetland Eco-restoration, China University of Geosciences (Wuhan). Anions (SO_4_^2−^, NO_3_^−^, Cl^−^, and F^−^) were measured using ion chromatography (IC; DX-120, Dionex, Sunnyvale, CA, USA), while cations (K^+^, Ca^2+^, Na^+^, and Mg^2+^) and certain minor elements (Si, Fe and Sr^2+^) were determined via inductively coupled plasma atomic emission spectrometry (ICP-AES, iCAP 6300 Duo View ICP-AES spectrometer, Thermo Electron Corporation, Waltham, UK). The wave numbers of K^+^, Ca^2+^, Na^+^, Mg^2+^ Si, Fe and Sr^2+^ were 766.490, 422.673, 589.592, 285.213, 251.661, 259.940 and 407.771, respectively. Ionic balance errors were within ±10%. The isotopic *δ*^18^O and *δ*D were measured with an ultrahigh-precision isotopic water analyzer (L2130-I, Picarro Inc., Santa Clara, CA, USA). These values were expressed in *δ* per milliliter relative to Vienna Standard Mean Ocean Water (V-SMOW), with precisions of 0.025‰ and 0.1‰, respectively. Trace element analysis was performed on an inductively coupled plasma mass spectrometer (ICP-MS, 7500a, Agilent, Santa Clara, CA, USA) at the State Key Laboratory of Geological Processes and Mineral Resources, China University of Geosciences (Wuhan). ^115^In was used as an internal standard.

### 2.3. Sediments Sample Collection and Analysis

**Figure 2:**
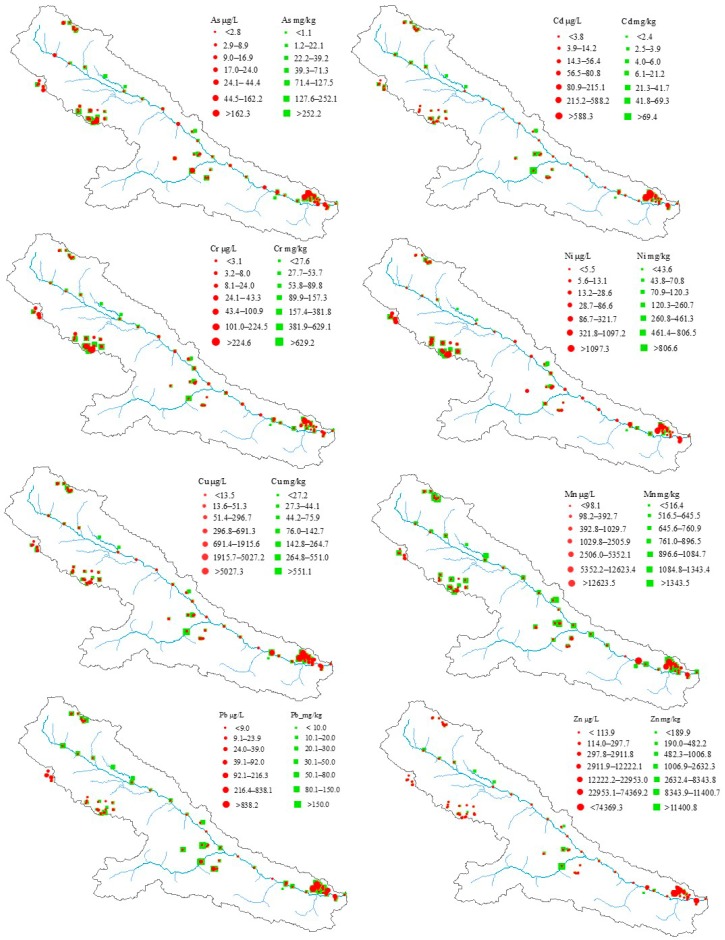
Distributions of heavy metals in surface sediments and waters. Green squares describe levels of HMs in sediment (units: mg/kg), and red circles describe HMs in water (units: μg/L).

As sediments contain a historical record of the natural and anthropogenic fluxes of HMs input into the water basin [28], a total of 104 surface sediment samples (0–20 cm in depth) were collected from locations close to sites from which water samples were collected (listed in Table 1 and shown in Figure 2) to analyze the sources of HMs. According to the method described by Bu et al. [10], with a clean plastic dustpan and brush, approximately 1 kg of fresh sediments was stored in plastic bags. All samples were air-dried at ~20 °C; sorted through a 2 mm plastic sieve to remove large plant roots, gravel-sized stones, and other debris; ground and homogenized with an agate mortar; and passed through a 200-mesh sieve.

A series of parameters including the concentration of the eight HMs, sediment total organic carbon (TOC), and pH were determined to better understand the spatial variations and their associations with different metals in the area [29].

Each sample (0.1 g) of milled soil was collected and placed in a polypropylene vessel and mixed with 2 mL of concentrated HNO_3_ and 1 mL of HClO_4_. The solution was heated on an open hot plate for approximately 4 h until white fumes were given off, and then the residue was re-dissolved in a plastic bottle with 2 mL of 4 mol L^−1^ HCl and diluted to 10 mL with deionized water. The concentrations of the eight HMs were measured via inductively coupled plasma mass spectrometry (ICP-MS, 7500a, Agilent, Santa Clara, CA, USA) at the State Key Laboratory of Geological Processes and Mineral Resources, China University of Geosciences. The national standard reference samples GSS-1 and GSR-1 were used for quality control of the analyses. The corresponding relative standard deviation (RSD) values were less than 5%. Total organic carbon in sediment (as a percentage) was measured using a TOC analyzer (VCPH, Shimadzu, Kyoto, Japan).

The pH of the sediments was measured in 1:2.5 sediment:water. The suspension was left to stands overnight before pH determination. The pH was measured using a pH meter (D-52, Horiba, Kyoto, Japan) with the calibration of pH 4, pH 7 and pH 9 standards. For electric condutivity (EC) determination, sediment (5 g) was taken in 50 mL polypropylene tubes, to which distilled water (30 mL) was added. The lid was closed tightly, and the tube was shaken for 5 min. Subsequently, EC was measured using an EC meter (D-52, Horiba, Kyoto, Japan).

### 2.4. Factor Analysis

Factor analysis (FA) is a conventional multivariate statistical method that determines the general relationship between measured variables by revealing multidimensional patterns that may be useful for classifying the original data [30,31,32,33]. FA is used to explore the possible sources and the hydrogeochemical processes affecting HMs by reducing the dimensionality of the dataset to several influencing factors. In this study, we used SPSS, version 19.0 (SPSS Inc., Chicago, IL, USA), for this analysis.

## 3. Results and Discussion

### 3.1. Water Quality Assessment

Water contamination by HMs (As, Mn, Cr, Ni, Cu, Cd, Pb, and Zn) was evaluated by comparing their concentrations with the guidelines for drinking water and surface water established by the China Environment Protect Agency (EPA) in 2002 and 2006 (Table 2). Because the surface water quality in this headwaters region plays an essential role in the supply of water resource, Grade I of Chinese surface water standards (GB 3838-2002) [34] served as the primary guideline value against which to evaluate the HM (As, Cr, Cu, Cd, Pb, and Zn) contamination. For Mn and Ni, the guideline values provided by the Chinese drinking water standards (GB 5749-2006) were used [35]. Mineral leached water and pond water were not evaluated, because there was no direct hydraulic connection between these points and the Heihe River, although the samples contain high levels of HMs.

As shown in Table 2, the results of water quality in the headwaters region of the Heihe River, all HMs in the main course were within the corresponding guideline values, indicating that the water quality of the main course of the Heihe River is good and without HM contamination.

However, in the investigated tributaries, the mean concentrations of seven HMs (excluding Arsenic) exceeded the limit recommended by the Chinses surface water and Chinese drinking water standards to varying extents (shown in Table 3). The mean Cr concentration was 1.29 and 4.29 times higher than the Grade I standard of Chinese surface water in tributary 3 and tributary 4, respectively. Ni exhibited a similar trend, with mean concentrations 1.31 and 8.19 times higher than those of the drinking water standard in tributaries 3 and 4. This result indicates that water in tributary 4 was severely contaminated with Cr and Ni. The mean manganese concentration was 1.68 times higher than the drinking water limit value in tributary 6. This result might not reflect the influence of Mn pollution due to the inadequate samples sizes. Moreover, the mean concentration of Pb was 1.36, 2.81 and 1.28 times higher than the Grade I standard for surface water in tributary 3, tributary 5, and tributary 11, respectively. For Cu, Cd, and Zn, the most severely contaminated stream was tributary 11, which was located within the Zhamashi polymetallic belt. Mean concentrations of Cu, Cd, and Zn in this tributary were 1.91, 2.49 and 9.7 times higher than the Grade I standards for Chinese surface water respectively. In general, Cr and Ni contamination mainly appeared in the upper reaches, whereas Cu, Cd, and Zn contamination primarily appeared in lower reaches. However, Pb contamination was found in different reaches of this study area. Except for Arsenic and Mn, other six HMs in the water of the tributaries appeared to be influenced by mining activity, shown in Table 1.

### 3.2. Distribution Characteristics of Heavy Metals in Sediment and Water

Compared with the mean background sediment metal concentrations in the Heihe River basin [10], except for Mn, the remaining seven HMs in sediments show apparent enrichment and considerably greater spatial variability (Figure 2 and Table 4). This result indicates that mining activity has significantly increased the seven HMs in surface sediments. The mean concentrations of these HMs (As, Cr, Ni, Cu, Cd, Pb, and Zn) are approximately 2, 1.5, 1.5, 2, 9, 12.5, and 14 times higher than the background mean values given by Bu et al. [10] respectively, while the medians are slightly less than the background mean values, as shown in Table 4. These results show that a small number of sediment samples detected extremely high levels of HMs. We preliminarily understand the order of contamination to be Zn > Pb > Cd > Cu = As > Cr = Ni > Mn.

The water quality results described above reveal different degrees of HMs contamination in several tributaries. However, the leaching of some minerals into steam water and pond water, which was therefore present a relatively high HM concentration, was not considered. In Figure 2, all water samples plotted.

The above water quality assessment shows that there was no arsenic pollution in any of the tributaries or the main course of the Heihe River. However, Figure 2 shows a large range of arsenic concentrations. In addition, the arsenic concentration in both sediment and water exhibited relatively high concentrations at the ore concentration belt of Yushigou and Zhamashi. High concentrations of Cr and Ni in sediments and waters were mainly found in the Reshuigou and Yushigou areas (Figure 1b and Figure 2), where the Yushigou ophiolite belt is located (shown in Figure S1). These results indicated that the sources of Cr and Ni might be the Yushigou.

The mean manganese (Mn) concentration for sediments (770.65 mg/kg) was slightly lower than the background value (818.84 mg/kg) described by Bu et al. [10]. Moreover, the majority of water samples showed a low Mn concentration (<100 μg/L), except for eight mineral leached waters with a high concentration (>1400 μg/L) collected in polymetallic or Mn mines around Zhamashi (Figure 1b and Figure 2). The consistent pattern of low Mn both in waters and sediments but high Mn in the waters and sediments around the corresponding mines might suggest that there were no apparent effects related to the distribution of the Mn mines.

The HM concentrations of Cu, Cd, Pb and Zn samples were mainly collected in the Chuancigou and Zhamashi areas, which included the polymetallic and lead–zinc deposits. HMs in waters produced a similar spatial distribution pattern, and relatively high values (including the peak values) of the four HMs (Cu, Cd, Pb, and Zn) were found in the Zhamashi polymetallic belt. Based on the above analysis, except for Mn, the spatial distribution of HMs in waters and sediments shows a high degree of consistency and is coincident with the mine areas. The strongly consistent distribution of HMs in waters and sediments reflects a close connection between water and surface sediment. Our sampling sites, such as SC, HC, YS, ZY, DY, and QF, are located near the uppermost sections of tributaries at high elevations, as shown in Figure 1b. In nature, weak weathering is not conducive to the enrichment of heavy metals in surface sediments and water bodies [36]. Thus, mining activity might increase the levels of HMs in sediments by accelerating bedrock weathering [10,37], especially with the open-pit mining method practiced in Yushigou and Chuancigou (Table 1). The above analysis explains the high concentrations of Cr, Ni, and As in the Yushigou area. However, the explanation for Cr, Ni, and As cannot be applied to analyze the high concentrations of Cu, Mn, Pb, Zn, and As in Zhamashi because underground mining was the primary practice in Zhamashi.

### 3.3. The Interaction of Surface Water, Groundwater, and Mining Waste Leaching Water

**Figure 3:**
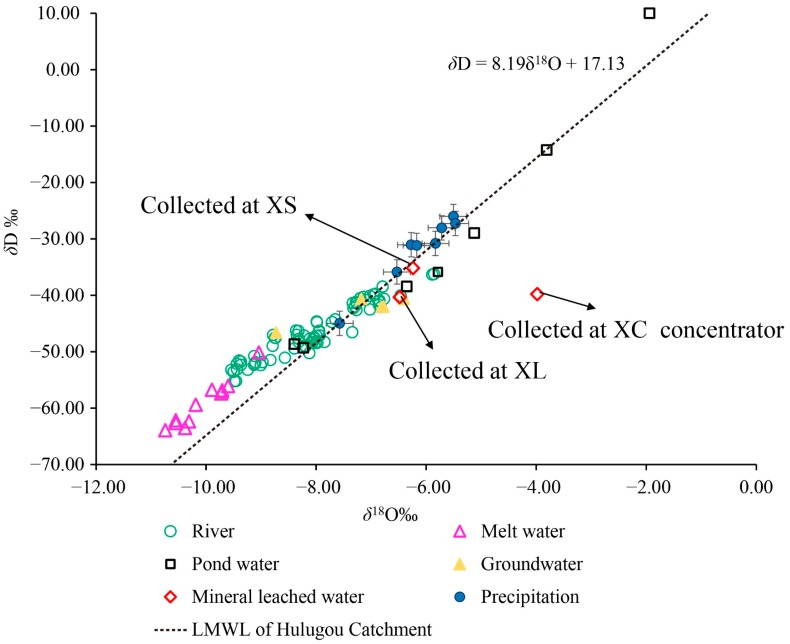
The dual isotope plot of *δ*D vs. *δ*^18^O for different types of water in 2013 compared to the local meteoric water line for Hulugou Catchment. LMWL represents the local meteoric water line of Tong et al. [38].

Analyzing the processes of basin runoff aid in understanding the pathway of HM transport. The pattern of *δ*D and *δ*^18^O in all water bodies were analyzed. The mean isotopic compositions of precipitation at different elevations were adopted as reference values [6,38]. Figure 3 shows the clustering of data for meltwater, precipitation and mineral leached waters. As shown in Figure 3, the locations of *δ*^18^O versus *δ*D for river water, groundwater, pond water and mineral leached water in the different elevation zones were close to or offset from the local meteoric water line (LMWL) [38], suggesting a relatively shallow depth for the basin hydrological cycle in the headwaters of the Heihe River.

Compared with other waters, meltwaters had lower values of *δ*^18^O and *δ*D than those found in the other water bodies in the headwaters of the Heihe River. All river water isotope compositions plotted between the cluster of melt water and the cluster of precipitation, meaning that precipitation and glacier meltwater co-supplied the river during August 2013.

Above an elevation of 4000 m, more negative *δ*^18^O values for river waters were presented, ranging from −9.54‰ to −8.08‰, which suggested that the upper reaches of rivers mainly recharged from glacier melt. However, as elevation decreased, the river water isotope composition gradually approached the LMWL and the average isotopic composition of precipitation. At elevations below 4000 m, relatively positive *δ*^18^O values ranged from −7.80‰ to −1.94‰, indicating that precipitation is the main water source in middle to lower reaches of this study area.

As the study area is a typical nested watershed model, with the decrease in elevation, the river catchment area gradually increases, and meltwater and precipitation discharge into the Heihe River along the way. The isotopic composition contribution from precipitation to river water increased, as expected. This pattern agrees with previous research results [6,12,39]. Moreover, Gao et al. [12] discovered that the contribution ratio of glacier meltwater to river discharge was quite low in summer due to the relatively small glacier (less than 1% of the basin area) and high level of precipitation.

Groundwater was primarily recharged from meltwater in the upper reaches, and the ratio of precipitation to meltwater increased as elevation decreased. The *δ*^18^O and *δ*D values in mineral leached water collected from the abandoned mine pit at XL and the bottom of the ore pile at XS were close to the LMWL [38] and between the values for groundwater and precipitation. This result suggests that the mine leached waters were co-supplied by precipitation and groundwater. The average isotope composition of the pond water was even higher than the mean level in precipitation at elevations of 2900–3000 m. These results indicate that pond water was recharged by precipitation and experienced strong evaporation.

The isotopic composition in river water should be particularly close to the cluster of precipitation isotopic compositions when precipitation directly recharges river water. However, mean δ^18^O values increased in the order of meltwater, river water, groundwater, mineral leached water and pond water and were −10.11‰, −8.07‰, −7.45‰, −6.36‰ and −0.23‰, respectively. Compared with pond water, mineral leached water, groundwater, and river water had lower values. This result also supported the conceptual model of the hydrologic process described in [6,12] indicating that precipitation reached the ground through a series of transport flows such as flow through the sediments, surface flow, and base flow entering the river [6,12,13,40]. Meltwater also infiltrated into the sediment aquifer and then through groundwater runoff discharge into river runoff [39,41,42].

### 3.4. Variability of Heavy Metals Along the Main Course

**Figure 4:**
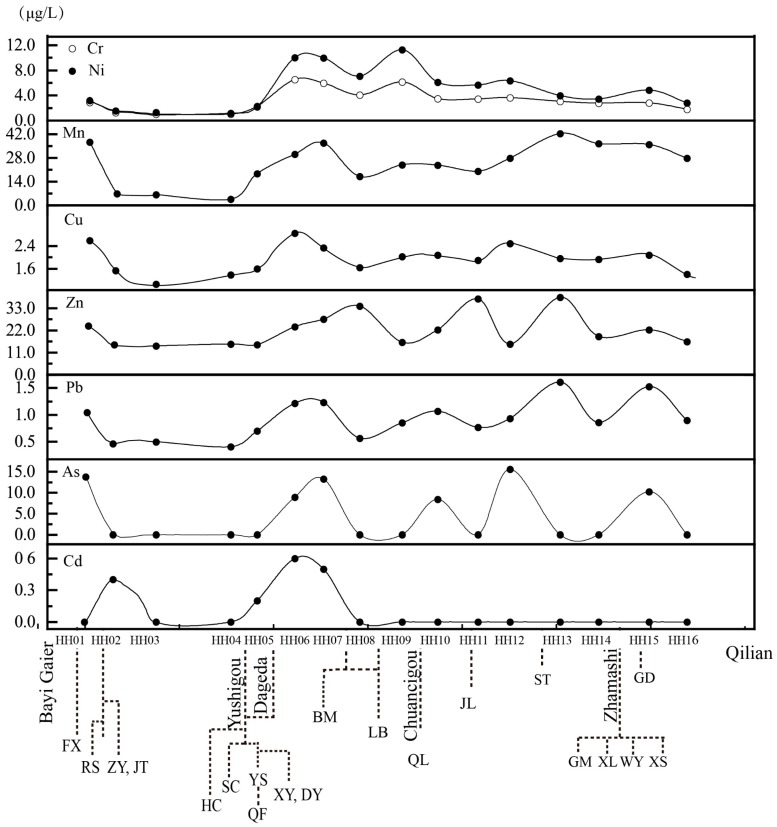
Evolution of HMs along the main courses of the Heihe River, indicating mines discharging into the river. HH01-HH16 indicates the 16 sampling sites in the main course. Different levels of tributaries are also marked (shown Figure S1).

The HM concentrations of 16 water samples (HH01–HH16) along the main course, corresponding to tributary (shown in Figure 1) discharge points, were used to further reveal the HM sources and transport routes (Figure 4) due to surface runoff bringing HMs into the main course from tributaries. Based on the isotope analysis results, the river runoff gradually increased along the main course. If the mines had not released the HMs via runoff, all eight HMs would be diluted by freshwater recharged from the precipitation and glacier melt in summer. This process explained why the concentrations of all eight HMs decreased at sampling site HH16. Moreover, as shown in Figure 4, the wave crests were often accompanied by the entry of tributaries affected by mining activities. Seven HMs were measured at sampling site HH01, not including Cd, indicating that the HM mineral sources were related to tributary 1. This area is located on the Zoulang Xibeipor iron belt, and the open-pit mining of the FX limestone might accelerate the weathering of the iron-bearing bedrock.

A series of Cd measurements was conducted at sampling site HH06, where six metals were grouped into three groups due to their similar variations. Group I comprised Cr and Ni; Group II included Mn and Cu; and Group III consisted of Pb, Zn and As. The variation in Cr and Ni exhibited the same patterns across the whole range; the two peaks at HH06 and HH09 reflected the exploration of ophiolite-related deposits in Yushigou and Chuancigou, respectively. In Group II, similar behavior of Mn and Cu was observed until sampling site HH13, because the manganese mine at ST significantly increased the Mn concentration at HH13. In Group III, Zn was observed to change more slowly than Pb after sampling site HH08; this behavior suggests that extra zinc was discharged into the main course before sampling site HH08. The corresponding tributaries (Figure 4) suggest that BM and QL might be the mineral sources of zinc. For arsenic, a similar fluctuation pattern is shown in Figure 4, but with a more sensitive response to the inputs into tributary 1, tributary 4, tributary 6, and tributary 11 (shown in Figure 1 and Figure 4).

### 3.5. Heavy Metal Provenance Tracing (Factor Analysis)

Factor analysis (FA) was performed to distinguish the variability among eight heavy metals in sediments and to verify the potential mineral sources in our study area. Table 5 displays the factor loadings with a VARIMAX rotation, as well as the eigenvalues.

From the rotated component matrix for the FA (Table 5), five factors with eigenvalues exceeding one were extracted, accounting for 85.558% of the total variance. High positive loadings of Cd, Cu, Pb, and Zn in Factor 1 (F1) explained the highest amount of variance at 29.447%. Factor 2 (F2) explained 17.877% of the total variance, where Cr and Ni had high loading. Factor 3 (F3) accounted for 14.239% of the total variance, in which arsenic was positively related to Cu and Pb with high loading. Moreover, Facor 4 (F4) explained 13.509% of the total variance, which was characterized by favorable loadings for TOC and negative loadings on pH. Factor 5 (F5) contributed 9.181% of the total variance and was characterized by positive loadings for Mn.

Cu, Pb, and Zn have high affinities to polymetallic deposits and combinations of Cu–Pb–Zn and Cu–Zn are widespread [9]. In addition, Cd is considered a typical chalcophile element and is often found in Zn sulfides [24,43]. These metals are commonly found together in various types of ore deposits in this area [44], reflecting an adequate mineral source. Mining activity also plays an important role according to the strong enrichment intensity. Another chalcophile element, arsenic (As) is grouped into F3 with Cu. In F2, Cr and Ni might originate more from the underlying bedrock weathering but might be slightly affected by mining activity considering their relatively weak enrichment intensities. The elements are supplied by basic and ultrabasic rocks such as peridotite, basalt, and gabbro in the Yushigou and Chuancigou ophiolite sequences [19,20,44]. F4 can be treated as the influence of the acid mine discharge (AMD). There are definite loadings on Cd, Cu, Pb and Zn in F4, with negative loadings on Cr, Mn, and Ni. This pattern indicates that AMD transports HMs into the surface sediment. Mn is always treated as a conservative metal due to its stable physicochemical characteristics [45,46,47]; these features distinguish it from the other HMs [48].

### 3.6. Identifying Hydrogeochemical Processes

Factor analysis of the 34 variables was applied to reveal the influence of HMs released into water environments. The first six varimax rotated factors were extracted according to the Kaiser criterion (Table 6), accounting for 81.33% of the total variance. High positive loadings on As, Be, Co, Sn, Bi, Th, U, EC, F^−^, SO_4_^2−^, Fe, K, and Si in F1 explained the highest amount of variance at 34.60%, indicating that aluminosilicate weathering was the primary source for these elements [49]. The pH had a negative association and SO_4_^2−^ was positively related to these trace elements, indicating that the “H^+^” acid released during mining activities enhanced the release of those elements. Mining activities accelerated this process, especially in sulfide deposits as commonly reported [43,50,51].

F2 accounted for 10.88% of the variance, where Li, Mo, HCO_3_^−^, Ca, Na, and Sr^2+^ had positive associations; pH was also had a negative loading (−0.42) in this factor, reflecting the dissolution of post-Devonian carbonate or intermediate volcanic rocks. F3 accounted for 10.43% of the total variance, with definite loadings on EC, Rb, CO_3_^2−^, Cl, and Mg. This element combination indicated the dissolution of evaporites or rocks bearing layered silicates (e.g., hydromica, chlorite, glauconite) [20,44].

F4 was characterized by positive loadings on Mn, Cs, and NO_3_^−^, which explained 9.77% of the total variance and suggested artificial contamination [24,36]. Positive NO_3_^−^ is often related to the presence of organic matter or nutrients, reflecting the influence of anthropogenic activities such as agriculture and grazing. In our study area, grazing might more important than agriculture, as the alpine meadow pastures are widespread, whereas the total area of several farmlands near HH05 is so small (less than 4%), shown in Figure S2. Mn and Cs have robust biological affinities. Bacteria, terrestrial plants, and crops are enriched in these two elements [24].

F5 was responsible for 9.24% of the total variance and had strong positive loadings on Cu (0.843) and Zn (0.933). Pb also showed relatively weak affiliation with these two elements with a value of 0.385. This association could be attributed to Cu–Zn and Cu–Pb–Zn mining activity.

F6 accounted for 6.41% of the total variance, and Ni and Cr were strongly associated with high loadings of 0.94 and 0.91, respectively. Mixed sources originated from both natural and anthropogenic inputs while Cr and Ni were homologous and sourced from the weathering of basic gabbro and serpentine in ophiolites [24,36,43].

**Figure 5:**
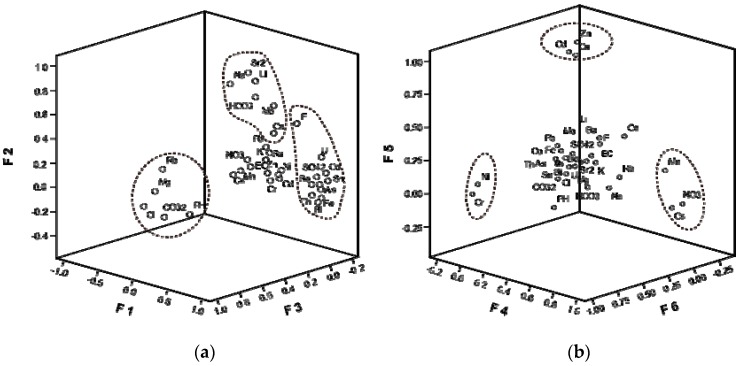
3-D plots of the FA loadings. (**a**) Weathering factors described by F1, F2, and F3. (**b**) Anthropogenic influences or inputs explained by F4, F5, and F6.

Based on the preceding water and sediment FA, we can recognize that mineral weathering and dissolution dominate the main hydro−chemical composition. HMs in water environments also arise from anthropogenic inputs such as mining, agriculture, and grazing. In general, F1, F2, and F3 mostly represent rock/mineral weathering, although they may be affected by mining activity, while F4, F5, and F6 explain the anthropogenic influences or inputs. Among these factors, mining might have a significant contribution, as explained by F5 and F6 (shown in Figure 5).

### 3.7. Impact of Mining Activity

To further understand the impact of mining, the mine investigation information was combined with the above analysis of HM mineral sources, water sources, and runoff paths. In both sediment and water, there are two ways that mining impacts the hydro−chemical conditions: enhanced mineral weathering and AMD. In the upper reaches of our study area, meltwater is a primary water source, and open-pit mining was the primary mining practice at several mines (RS, HC, XY, SC, YS, QF, and DY), which are mainly active in the Ophiolite Complex (shown in Table 1 and Figure S1). Open-pit mining activity apparent increases the contact between ore and oxygen by crushing ores and results in changes to the original redox state of the ore. When Meltwater though the mining area surface sediment, HMs of Cr, Ni, and Arsenic were carried to runoff. Considering the result of FA, AMD might also infiltrate into the sediment aquifer and promote HM dissolution from sediment minerals by providing “H^+^” [52,53,54].

A similar process also occurred at site near underground mining. Underground mining introduces oxygen to the deep geological environment and brings minerals to the surface to be deposited in spoil tips [45,51,55]. HMs in surface sediment can also be enhanced by mineral weathering. However, the weathering rate was far slower than that in open-pit mining because open-pit mining provides a larger contact area between the ore and the air. Table 1 shows that underground mining mainly is practiced at the mining sites in middle and lower reaches of this study area where precipitation is the main water source, and the rate of weathering is faster than that in upper reaches at elevation above 4000 (m.s.l).

Therefore, high level of HMs in the Zhamashi sites such as GM, WY, XL, and XS might be attributed to a comprehensive process. Precipitation leaches HMs from mineral spoil tips and surface sediment and transport these elements via runoff. AMD might not only enhance weathering and dissolution by reducing sediment pH, but also carry HMs into runoff directly. For the polymetallic mines at high elevations such as JT, meltwater is the primary source. Meltwater, as a primary carrier of HMs, directly pours into the river through underground seepage and surface runoff. HMs are mainly carried by AMD.

## 4. Conclusions

This research investigated the spatial distribution of heavy metals (HMs) and assessed water quality in the headwater region of the Heihe River, which has a fragile ecological environment. Factor analysis (FA) and the isotope relationships of *δ*D and *δ*^18^O were used to identify the mineral sources and water sources. On this basis, the pathways of HMs were also recognized.

The results of water quality analysis suggested that tributaries were affected by mining activity and that excessive elements in surface waters had direct relationships with local mining areas. The ores varieties in the local mining areas were often composed of elements exceeding water quality standards. The consistent distribution of HMs in waters and sediments reflected a close connection between water and surface sediments. The different sources were distinguished based on FA in waters and sediments. HMs in sediments were inherited from the underlying parent bedrock. Mineral weathering and dissolution dominated the main hydro−chemical composition. HMs in water environments are mainly sourced from anthropogenic activities such as mining, agriculture, and grazing. Cr, Ni, Cd, Cu, Zn, As and Pb appeared to be influenced more by mining activity. Cd, Cu, Zn, As and Pb were sourced from the mining of metal sulfide deposits such as polymetallic, lead–zinc, manganese and iron mines. Cr and Ni were homologous and were sourced from the mining activity in basic gabbro and serpentine of the ophiolites complex. Mn appeared to be influenced primarily by artificial activities such as agriculture and grazing.

Combining the mine investigation information with the above results on the water quality mineral sources, water sources, and runoff paths revealed, two main mining influences depending on different mining methods. For open-pit mining, mining promoted the release of HMs via enhanced weathering. For underground mining, HMs might have contributed more AMD at high elevations. As the elevation decreased, precipitation increased; A series of complex hydrological factor significantly affected leaching and runoff.

## Figures and Tables

**Table 1:** General information for sampling sites. The detailed information on water and sediment samples is presented. The several tributaries are also divided into different reaches of the headwaters region of the Heihe River basin.

Reaches	ID ^a^	Mine ^b^	Date	Mine Area (km^2^)	Ore Type	Elevation (m)	Mining Method (OP/UG) ^c^	Water Sample Collected from Several Mines						Sediment
								Meltwater	River Water	Groundwater	Pond Water	Mineral Leached Water	Total	
Upstream	1	FX	20/August	0.087	Marble	4280–4582	OP	1	1			3	5	5
	2	JT	19/August	1.115	Polymetallic	3920–4100	UG		2		1		3	2
		ZY	19/August	0.498	Iron ore	4300–4600	OP		4	1			5	3
	3	RS	18/August	0.570	Iron ore	4190–4290	OP	4	5				9	3
	4	HC	21/August	0.105	Serpentine	4238–4308	OP		3			1	4	3
		XY	21/August	0.070	Serpentine	4200–4330	OP		2				2	1
		YS	22/August	0.056	Chromite	4360–4539	OP	5		1			6	1
		SC	23/August	1.299	Asbestos	4362–4630	OP		3			2	5	6
		QF	23/August	0.313	Chromite	4280–4560	OP		2			1	3	2
		DY	24/August	0.034	Serpentine	4220–4290	OP		3	1			4	3
Middle-stream	5	BM	25/August	0.107	Manganese	3620–3650	UG		1				1	5
	6	LB	26/August	0.523	Lead–Zinc	4020-4460	OP/UG		2				2	1
	7	QL	25/August	0.520	Iron ore	3520–3959	UG		3				3	7
	8	JL	15/August	0.121	Polymetallic	3920–4100	UG		3		1	1	5	4
	9	XC	26/August		Ore dressing							1	1	3
Down-stream	10	ST	14/August	0.160	Manganese	3018–3130	UG		4			1	5	3
	11	WY	8/August	0.189	Polymetallic	2790–3150	UG		6	1	1	1	9	5
		XL	9/August	0.117	Polymetallic	3084–3460	UG		2	1		1	3	2
		XS	10/August	0.540	Polymetallic	3150–3680	OP/UG		6	1		3	10	10
		GM	11/August	0.134	Polymetallic	3000–3530	UG			1	3	4	8	4
		XG	12/August	0.195	Polymetallic	2730–3159	UG		4				4	8
	12	GD	13/August	0.550	Polymetallic	3760–4030	UG		2	1		3	6	5
		HH	27, 28/August						16				16	18
Total	14					104		10	74	7	6	22	119	104

Space indicates no corresponding information is provided. ^a^ The serial number of the tributary catchment investigated along the Heihe River. ^b^ all mine locations in Table 1 have been defined in Section 2.1.2 and Figure 1; HH-main course of the Heihe River. ^c^ UG-underground mining; OP-open pit mining.

**Table 2:** Chinese national quality standards for drinking water and surface water quality (units: μg/L).

Water Standard	As	Mn	Cr	Ni	Cu	Cd	Pb	Zn
Grade I ^a^	10	–	10	–	10	1	10	50
Drinking ^b^	10	100	10	20	1000	5	10	1000

(–) Indicates no information is provided; ^a^ Chinese surface water standards (GB 3838-2002) [34]. Grade I: Clean water from headwater and national conservation area that can be used for domestic purposes after simple disinfection, for recreational purposes and irrigation; ^b^ Chinese drinking water standards (GB 5749-2006) [35].

**Table 3:** Water quality assessment of heavy metals (HMs) in tributaries and the main course. Mean concentrations of HMs in serval tributaries (units in μg/L).

Tributary ID	Mines ^a^	Samples ^b^	As	Mn	Cr	Ni	Cu	Cd	Pb	Zn
1	FX	2	0.00	40.02	2.18	3.03	2.56	0.10	2.92	25.70
2	ZY, JL	7	1.51	11.68	1.83	2.15	1.20	0.19	1.63	38.31
3	RS	9	4.12	58.06	12.86	26.26	4.26	0.38	13.61	28.23
4	HC, SC, YS, QF, XY, DY	20	6.90	64.71	42.92	163.86	11.79	0.48	1.32	26.13
5	BM	1	0.00	2.65	1.02	1.69	3.56	0.50	28.15	2.74
6	LB	2	6.10	167.65	1.07	7.82	7.07	0.50	1.01	29.95
7	QL	3	2.13	66.61	7.19	8.54	3.72	0.83	1.57	23.28
8	JL	3	0.00	24.64	1.22	3.85	4.95	1.07	5.10	55.20
10	ST	4	3.38	16.94	0.95	4.45	7.76	0.75	2.89	33.17
11	WY, XL, XS, GM, XG	21	1.09	46.73	1.82	3.13	19.05	2.49	12.82	489.98
12	GD	3	2.53	36.72	2.51	14.67	4.42	0.43	1.69	25.40
	HH	16	4.39	24.62	3.27	5.00	1.92	0.11	0.91	22.26

Underlines indicate that the mean concentration of the HM exceeds the corresponding standard, as presented at the beginning of this section. ^a^ all mine locations in Table 3 have been defined in Section 2.1.2 and Table 1. ^b^ Number of water samples which selected for evaluation.

**Table 4:** Comparisons of HM concentrations in the sediment from the headwaters region of the Heihe River (units in mg/kg).

Metal	This Study				Background ^a^
	Min	Max	Median	Mean	Mean
As	1.12	728.03	19.73	42.57	21.60
Mn	293.21	2106.08	722.64	770.65	818.84
Cr	2.43	1148.47	45.46	88.39	57.29
Ni	8.96	1615.34	43.31	110.45	70.22
Cu	6.66	2497.69	43.98	103.28	56.38
Cd	0.2	2349.91	2.97	27.39	2.93
Pb	0	26,538.82	23.22	470.22	37.35
Zn	31.87	210,880.62	128.31	2460.93	178.68

^a^ The background value according to [10].

**Table 5:** VARIMAX rotated factor loading matrix for sediments. The numbers in bold indicate that the loads are above 0.5.

Variables	F1	F2	F3	F4	F5
Cd	**0.974**	0.033	0.064	0.131	0.021
Cr	0.071	**0.978**	0.034	−0.072	−0.005
Cu	**0.607**	−0.063	**0.693**	0.097	−0.009
Mn	−0.060	0.023	−0.117	−0.064	**0.901**
Ni	−0.042	**0.984**	0.048	−0.014	−0.018
Pb	**0.887**	−0.013	**0.425**	0.112	−0.023
Zn	**0.975**	0.030	0.085	0.129	0.016
As	0.160	0.123	**0.881**	0.012	−0.063
pH	−0.035	0.034	−0.279	**−0.879**	−0.096
TOC (g/kg)	0.276	−0.042	−0.299	**0.735**	−0.151
EC (μs/cm)	0.264	−0.128	0.279	0.326	0.400
Eigenvalues	3.239	1.966	1.710	1.486	1.010
% of Variance	29.447	17.877	15.544	13.509	9.181
Cumulative %	29.447	47.323	62.867	76.376	85.558

**Table 6:** VARIMAX rotated factor loading matrix for waters. The numbers in bold indicate that the loads are above 0.5.

Variables	F1	F2	F3	F4	F5	F6
As	**0.99**	0.02	0.01	−0.01	0.07	0.11
Cd	0.40	0.05	0.01	0.01	**0.89**	0.02
Li	0.21	**0.82**	0.11	0.16	0.11	0.07
Be	**0.97**	0.06	0.01	0.07	0.13	0.13
Cr	0.22	-0.03	−0.02	−0.02	0.03	**0.94**
Mn	0.40	0.08	0.01	**0.87**	0.22	0.05
Co	**0.96**	0.08	0.01	0.06	0.15	0.21
Ni	0.26	0.01	−0.07	0.01	0.10	**0.91**
Cu	0.31	0.08	0.01	0.08	**0.89**	0.04
Zn	−0.04	0.12	0.00	0.04	**0.96**	−0.03
Rb	−0.07	0.23	**0.76**	0.46	0.06	0.03
Mo	0.17	**0.56**	−0.08	−0.14	0.09	−0.07
Sn	**0.99**	0.03	0.02	−0.01	0.08	0.11
Cs	0.00	0.07	0.14	**0.94**	−0.04	0.07
Ba	−0.11	0.09	−0.21	−0.07	0.14	−0.37
Pb	0.07	0.20	−0.07	−0.09	0.16	0.02
Bi	**0.99**	0.02	0.01	−0.01	0.07	0.11
Th	**0.99**	0.02	0.01	0.02	0.07	0.11
U	**0.91**	0.27	−0.01	0.04	0.04	0.06
EC	**0.67**	0.26	**0.62**	0.22	0.17	0.04
pH	**−0.70**	-0.42	0.13	−0.16	−0.33	−0.03
CO_3_^2−^	−0.05	−0.16	**0.75**	−0.15	−0.12	−0.06
HCO_3_^−^	−0.12	**0.58**	−0.10	−0.13	−0.24	−0.32
F^−^	**0.61**	0.50	−0.01	0.27	0.26	0.01
Cl^−^	−0.02	−0.01	**0.96**	0.03	−0.01	0.07
NO_3_^−^	0.00	0.05	0.02	**0.95**	−0.03	−0.02
SO_4_^2−^	**0.96**	0.13	0.04	0.10	0.16	0.08
Ca	0.38	**0.40**	0.09	0.33	0.30	−0.16
Fe	**0.99**	0.03	0.01	−0.01	0.07	0.11
K	0.46	0.23	0.17	0.17	0.08	−0.06
Mg	0.11	0.13	**0.94**	0.09	0.06	0.06
Na	0.06	**0.81**	0.24	0.36	−0.04	0.03
Si	**0.98**	0.07	−0.01	0.02	0.08	0.15
Sr^2+^	0.07	**0.86**	0.09	0.15	0.10	0.00
Eigenvalues	11.77	3.70	3.55	3.32	3.14	2.18
% of Variance	34.60	10.88	10.43	9.77	9.24	6.41
Cumulative %	34.60	45.48	55.91	65.68	74.92	81.33

Extraction method: Principal component analysis. Rotation method: Varimax with Kaiser normalization.

## Supplementary Materials

The following are available online at http://www.mdpi.com/1660-4601/15/9/1987/s1, Figure S1: Geological map of the headwater regions of Heihe River Basin; Figure S2: The Land Use Coverage of the headwater region of the Heihe River Basin.
