# Supplementary material for: Effects of Mining Activities on the Release of Heavy Metals (HMs) in a Typical Mountain Headwater Region, the Qinghai-Tibet Plateau in China
Source: Int J Environ Res Public Health. 2018 Sep 12;15(9):1987. doi: 10.3390/ijerph15091987 (PMC6165501; doi:10.3390/ijerph15091987)
Supplement: Supplementary file 1 [file ijerph-15-01987-s001.pdf]

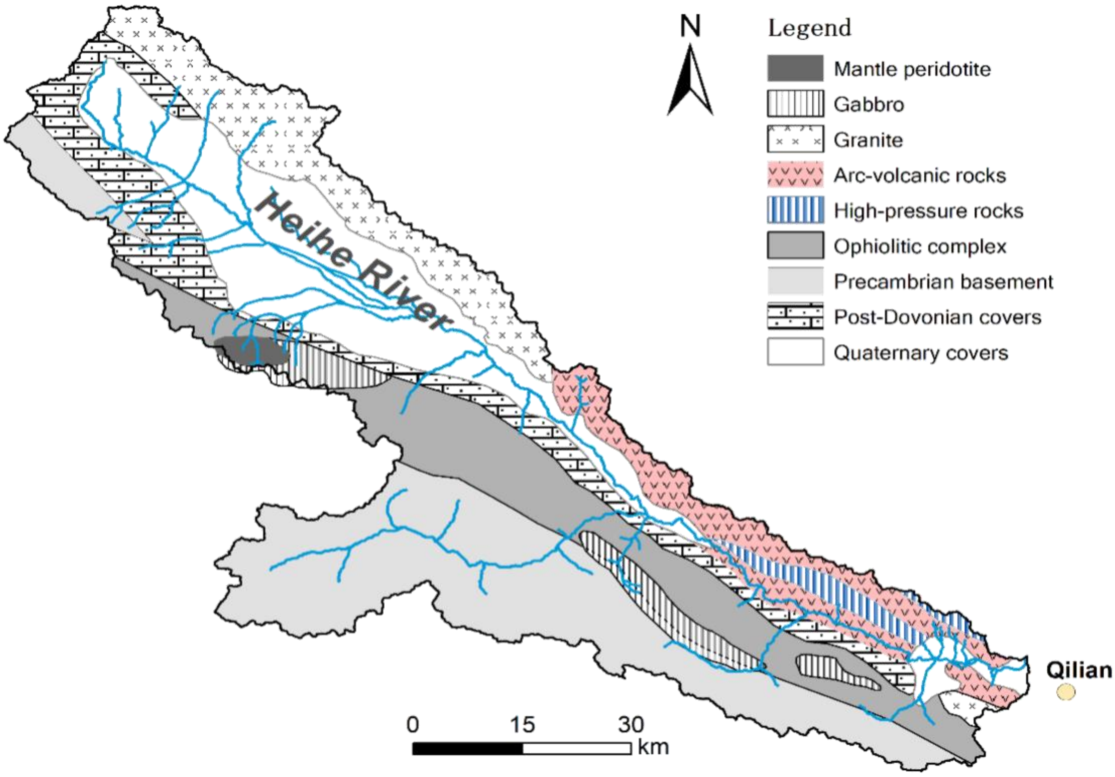

Figure S1. Geological map of the headwater regions of Heihe River Basin.

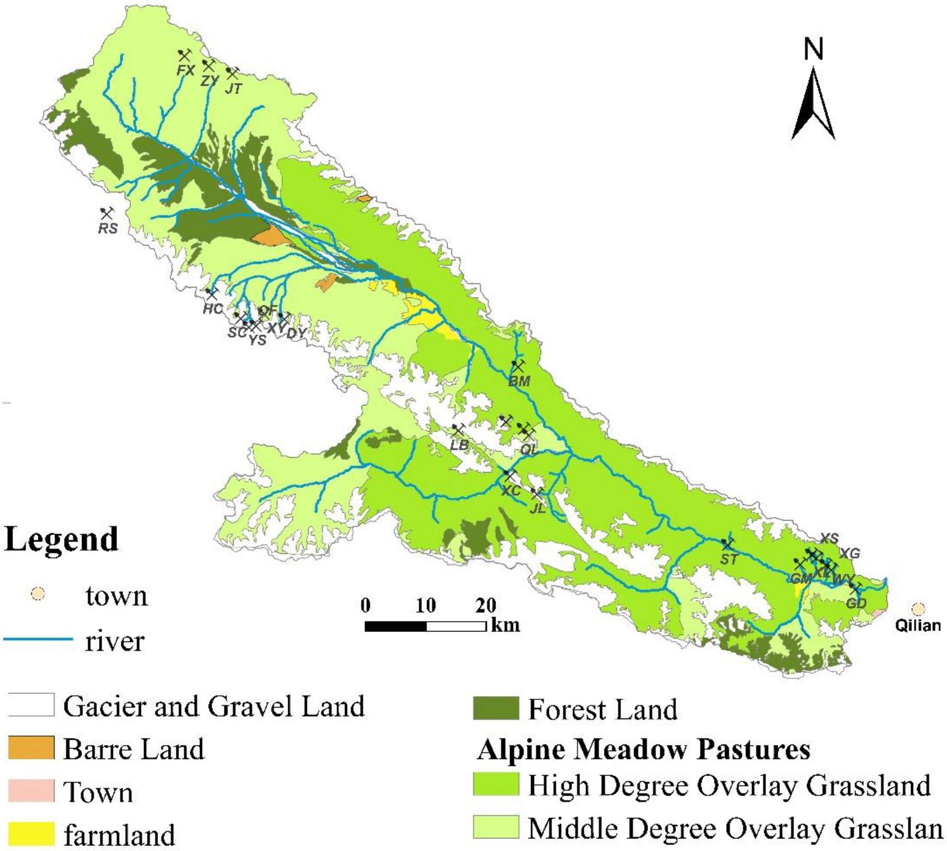

Figure S2. The Land Use Coverage of the headwater region of the Heihe River Basin.
